# Family dynamics and adolescent behavioral disorders: Insights from the NKI Rockland Sample

**DOI:** 10.1192/j.eurpsy.2025.846

**Published:** 2025-08-26

**Authors:** M. Grigoriou, J. von Trott, P. Lalousis

**Affiliations:** 1 Psychology, University of Limassol, Limassol, Cyprus; 2 Institute of Psychiatry, Psychology & Neuroscience, King’s College London, London, United Kingdom; 3 Department of Psychiatry and Psychotherapy, Ludwig-Maximilian-University Munich, Munich, Germany

## Abstract

**Introduction:**

Familial dynamics profoundly impact the behavioural development of adolescents. This study investigates the impact of family-related behaviours, such as hyperactivity, aggression, and callous-unemotional features, on social responsiveness and the propensity for behavioural disorders, offering essential insights for targeted interventions.

**Objectives:**

The study aimed to: (1) examine the influence of hyperactivity, aggression, callous-unemotional traits(CU), and thrill-seeking behaviour on social responsiveness and behavioural disorders; (2) assess the predictive efficacy of various behavioural traits through structural equation modelling (SEM) and logistic regression; and (3) recommend practical interventions derived from these findings.

**Methods:**

Data were obtained from adolescents 12 to 17 years participating in the enhanced Nathan Kline Institute Rockland Sample (NKI-RS), a continuous, institutionally focused initiative designed to establish a large-scale lifespan sample. Behavioural evaluations comprised the Behaviour Assessment System for Children (BASC), Social Responsiveness Scale (SRS), and Inventory of Callous-Unemotional Traits (ICU-P). Structural Equation Modelling (SEM) was utilised to investigate the intricate associations between behavioural characteristics and social responsiveness (Figure 1), whereas logistic regression was applied to forecast the probability of behavioural disorders (Figure 2).

**Results:**

Structural Equation Modelling analysis indicated that CU traits were the most significant predictor of diminished social responsiveness (β = 0.738, p < 0.001), succeeded by hyperactivity (β = 0.384, p = 0.069) and agression (β = 0.183, p = 0.038). Engagement in fun-seeking behaviour demonstrated a protective effect (β = -0.638, p = 0.001). Logistic analysis indicated that elevated ICU-P scores heightened the probability of behavioural problems (OR = 2.09, p < 0.001), whereas fun-seeking behaviour diminished this probability (OR = 0.52, p < 0.001), yielding an AUC of 0.83 (Figure 3).

**Image 1:**

**Figure:**
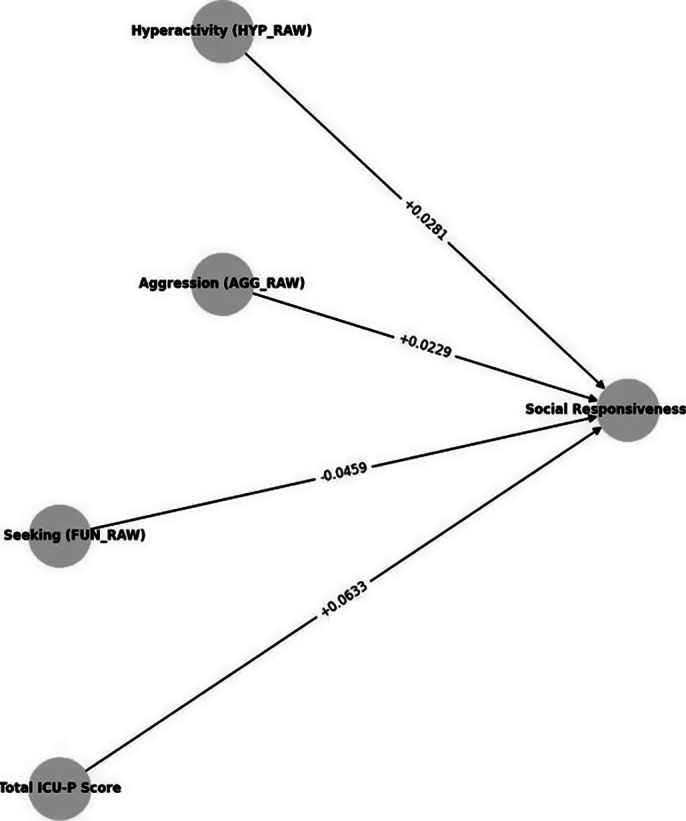

**Conclusions:**

CU qualities represent the most significant risk factor for behavioural disorders, whereas a propensity for fun-seeking behaviour offers a protective effect. Practical implementation should focus on two main areas: (1) early screening for CU traits and hyperactivity in family settings to identify at-risk adolescents, and (2) promoting adaptive fun-seeking activities that enhance social responsiveness and reduce behavioral risks. Family-oriented therapies that include good social activities and behavioural management can markedly diminish the risks associated with behavioural disorders.

**Disclosure of Interest:**

None Declared

